# Oculoplastic Interventions in the Management of Ocular Surface Diseases: A Comprehensive Review

**DOI:** 10.3390/life15071110

**Published:** 2025-07-16

**Authors:** Seyed Mohsen Rafizadeh, Hassan Asadigandomani, Samin Khannejad, Arman Hasanzade, Kamran Rezaei, Avery Wei Zhou, Mohammad Soleimani

**Affiliations:** 1 Department of Oculofacial Plastic and Reconstructive Surgery, Farabi Eye Hospital, Tehran University of Medical Sciences, Tehran 1417614411, Iran; mohsenraf1354@gmail.com; 2 Department of Ophthalmology, University of California San Francisco, San Francisco, CA 94143, USA; hassan.asadigandomani@ucsf.edu; 3 Student Research Committee, Shahid Beheshti University of Medical Sciences, Tehran 1983963113, Iran; saminkhannejad@gmail.com (S.K.); arman29h@gmail.com (A.H.); kamran_rezaei@yahoo.com (K.R.); 4 Bernice and Harvey Jones Eye Institute, University of Arkansas for Medical Sciences, Little Rock, AR 72205, USA; averyzhou24@gmail.com; 5 Department of Ophthalmology, University of North Carolina at Chapel Hill, Chapel Hill, NC 27599, USA

**Keywords:** dry eye syndrome, Steven-Johnson syndrome, toxic epidermal necrolysis, ophthalmology

## Abstract

This study aimed to comprehensively review surgical interventions for ocular surface diseases (OSDs), including dry eye syndrome (DES), exposure keratopathy, Stevens-Johnson syndrome (SJS), toxic epidermal necrolysis (TEN), and ocular graft versus host disease (oGVHD), and to highlight the indications, contraindications, outcomes, and complications of various oculoplastic procedures used in their management. A narrative review was performed based on expert-guided selection of relevant studies retrieved from PubMed, Scopus, and Web of Science. Relevant keywords included “ocular surface disease”, “dry eye syndrome”, “exposure keratopathy”, “thyroid eye disease (TED)”, “neurotrophic keratopathy (NK)”, “Stevens-Johnson syndrome”, “toxic epidermal necrolysis”, “punctal occlusion”, “tarsorrhaphy”, “botulinum toxin”, “eyelid loading”, “retractor weakening”, “corneal neurotization (CN)”, “amniotic membrane transplantation (AMT)”, “conjunctival flap”, “ocular graft versus host disease”, and “salivary gland transplantation (SGT)”. Studies addressing surgical approaches for OSDs were included. In conclusion, surgical options for OSDs offer significant benefits when non-invasive treatments fail. Surgical techniques such as punctal occlusion, eyelid fissure narrowing, AMT, and conjunctival flap procedures help stabilize the ocular surface and alleviate symptoms. Advanced methods like CN and SGT target the underlying pathology in refractory cases such as oGVHD. The outcomes vary depending on the disease severity and surgical approach. Each procedure carries specific risks and requires individualized patient selection. Therefore, a tailored approach based on clinical condition, anatomical involvement, and patient factors is essential to achieve optimal results. Ongoing innovations in reconstructive surgery and regenerative medicine are expected to further improve outcomes for patients with OSDs.

## 1. Introduction

Ocular surface diseases (OSDs) are a major cause of ocular discomfort and visual impairment, often presenting with symptoms such as pain, foreign body sensation, blurred vision, and photophobia. These conditions stem from diverse etiologies, including decreased tear production, meibomian gland dysfunction (MGD), or limbal stem cell deficiency (LSCD), particularly in Stevens-Johnson Syndrome (SJS) and Toxic Epidermal Necrolysis (TEN). Neurotrophic Keratopathy (NK), Thyroid Eye Disease (TED), and ocular graft versus host disease (oGVHD) are also important causes of OSDs, although their pathophysiology involves different mechanisms such as neurotrophic impairment, inflammatory exposure, or immune-mediated damage. Neurological factors—such as facial or fifth nerve paralysis and impaired tear pumping in critically ill patients—as well as trauma-induced eyelid damage and infections like Herpes Zoster, also contribute to OSDs by disrupting normal eyelid and ocular surface function [1].

Non-surgical management strategies for OSDs often focus on optimizing the natural tear film and preventing tear evaporation. Techniques such as using eyelid covers or performing a temporary tarsorrhaphy can be effective in patients with impaired eyelid closure [1,2]. Therapeutic scleral lenses have shown promise in managing exposure keratopathy, particularly in cases of severe OSDs [3]. Additionally, Prosthetic Replacement of the Ocular Surface Ecosystem (PROSE) devices have demonstrated efficacy in treating moderate-to-severe dry eye syndrome (DES). For SJS and TEN, conventional treatments include vitamin A eye ointment, artificial tears, anti-inflammatory medications, and removal of pseudomembranes. However, when conservative interventions fail, surgical intervention may be necessary [4,5,6,7,8,9].

This narrative review explores the role of various oculoplastic interventions in managing OSDs, along with their indications, contraindications, and associated complications, based on expert-guided selection of relevant literature.

## 2. Methods

This narrative review was conducted to comprehensively assess the role of surgical interventions in the management of OSDs. A thorough literature search was carried out using major electronic databases, including PubMed, Scopus, and Web of Science, without any restriction on the publication date. Only articles published in English were considered.

Keywords used in the search included: “ocular surface disease”, “exposure keratopathy”, “dry eye syndrome”, “Stevens-Johnson syndrome”, “toxic epidermal necrolysis”, “oculoplastic surgery”, “punctal occlusion”, “tarsorrhaphy”, “botulinum toxin”, “eyelid loading”, “eyelid retractor weakening”, “corneal neurotization (CN)”, “amniotic membrane transplantation (AMT)”, “conjunctival flap”, “thyroid eye disease”, “neurotrophic keratopathy”, “ocular graft versus host disease”, and “salivary gland transplantation (SGT)”. Search terms were applied individually and in various combinations to maximize coverage of relevant literature.

The selection process involved an initial screening of titles and abstracts to identify potentially relevant studies. Full-text articles were then assessed for inclusion based on their relevance to the surgical management of OSDs, with a particular focus on indications, techniques, outcomes, complications, and contraindications. In addition to peer-reviewed articles, the reference lists of key publications were manually reviewed to identify additional relevant studies.

As this is a narrative review, no formal protocol was registered, and no quantitative synthesis (meta-analysis) was performed. However, efforts were made to include a broad and balanced selection of studies to reflect the current state of knowledge. Illustrative clinical data and case examples were incorporated when available, in order to enrich the discussion with real-world insights and to highlight practical considerations for surgical decision-making. This review aims to consolidate current evidence and provide practical insights for clinicians treating patients with moderate-to-severe OSDs who have not responded to medical therapy.

## 3. Oculoplastic Interventions

### 3.1. Punctal Occlusion

Punctal occlusion increases tear retention on the ocular surface by temporarily or permanently blocking the lacrimal drainage system at the punctum or canaliculus [10,11]. With the invention of punctal plugs (PP) and canalicular plugs (CP) which block lacrimal drainage [12], the utilization of surgical methods has decreased [10]. They come in various shapes and materials, including temporary collagen plugs and semi-permanent options like silicone and polymers [12]. CPs, typically cylindrical, may be temporary or permanent and expand to occlude the canaliculus upon contact with tears [12].

Plugs help alleviate symptoms of DES by minimizing dependence on artificial tears. Plugs are also effective for DES caused by conditions like Sjögren’s syndrome, SJS, and post-refractive surgery [11,12]. Other indications include contact lens-associated dryness, superior limbic keratoconjunctivitis (SLK), and NK. Plugs also enhance the efficacy of topical medications by extending contact time, and can also serve as drug delivery systems [10]. Of note, perforated plugs serve an opposite purpose and are particularly useful in managing punctal stenosis by improving tear drainage [11,12].

PPs and CPs are contraindicated in patients with active ocular infection, inflammation, ectropion, lacrimal obstruction, or material allergy [11,12,13]. Complications include plug loss or extrusion (25–50% of cases within two years), with higher risks for smaller or superior plugs [10,11,12]. In a retrospective cohort study by Kim et al., the spontaneous loss rate of PPs was reported to be 58%, with factors such as smaller plug size and upper eyelid punctum location significantly contributing to the increased risk of loss [14]. Partial extrusion can irritate the ocular surface, causing conjunctivitis or keratitis [10,11,12]. Migration of plugs may lead to canaliculitis or dacryocystitis, requiring surgical intervention, and can also cause punctal or canalicular stenosis [10]. Other potential issues include epiphora, granulation tissue, discomfort, biofilm formation, and corneal epithelial defect (CED) [10,11,12].

PPs and CPs are often used as a non-invasive treatment for DES, but in cases where complications such as granuloma formation or recurrent plug extrusion arise, surgical punctal occlusion may be considered [15,16]. This approach should be reserved for moderate to severe cases of DES, particularly when less invasive treatments, such as artificial tears and plugs, have proven ineffective. Surgical punctal occlusion can be performed in a permanent or reversible manner, and can either completely or partially occlude the lacrimal drainage system [17].

Surgical methods for punctal occlusion can be classified into heat-induced injuries, transposition or removal of the punctum or canaliculus, and creating a mechanical barrier [17,18]. Common techniques include heat-based procedures like cauterization, diathermy, and laser, with punctal cauterization being the most frequently performed due to its simplicity and speed [17,18]. Punctal cauterization has demonstrated excellent long-term anatomical success without recanalization in patients with oGVHD [19]. The effectiveness of punctal cauterization is influenced by both the anatomical site and the depth of the cautery applied. Superficial cauterization has a higher risk of recanalization making it less effective compared to deep cauterization [17,18]. In a retrospective cohort study by Wang et al., which involved a study population of 80 patients with 171 puncta and a follow-up period of 27 months, the rate of recanalization following punctal cauterization was reported to be 21% [16].

**Figure 1:**
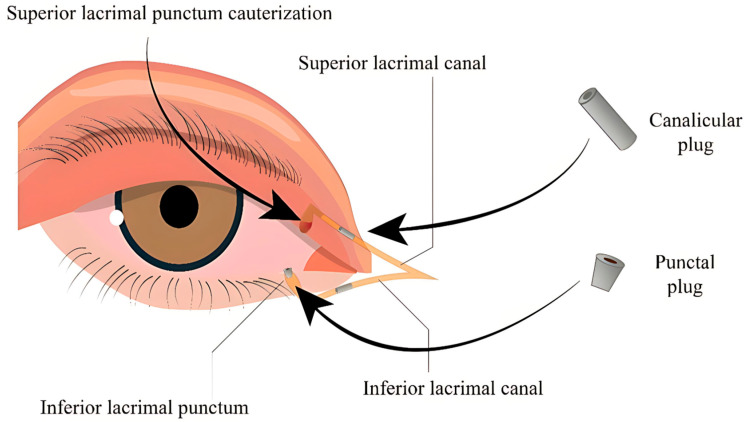
Techniques for occlusion of the lacrimal drainage system in the management of DES.

Notably, PPs, CPs, and cauterization are effective strategies for enhancing tear film retention in DES, especially when conservative treatments fail. While plugs offer a reversible and non-invasive approach, surgical occlusion methods like cauterization provide more durable outcomes in refractory or severe cases such as oGVHD (Figure 1).

### 3.2. Eyelid Fissure Narrowing Techniques

#### 3.2.1. Tarsorrhaphy

Tarsorrhaphy involves suturing the upper and lower eyelids together, either partially or completely, to close or narrow the interpalpebral fissure [20]. Tarsorrhaphy also protects the eyes from environmental exposures and infectious agents, and prevents friction injuries caused by eyelid movement [20,21,22,23]. Therefore, tarsorrhaphy aids in the healing process of the corneal epithelium and should be considered for patients with persistent CED, especially when medical and non-surgical treatments have failed [21]. This includes CEDs caused by infected ulcers, exposure keratopathy, DES, radiation keratopathy, ocular cicatricial pemphigoid (OCP), SJS, corneal melting, and NK [22,24]. Furthermore, tarsorrhaphy is beneficial in cases where there is a risk of corneal injury due to inadequate eyelid closure caused by facial nerve palsy, poor blinking, eye protrusion, ectropion, entropion, lagophthalmos, globe displacement, facial and eyelid burns, and following ophthalmic surgeries such as penetrating keratoplasty and blepharoplasty [20,22,25]. Tarsorrhaphy can be used in combination with other therapeutic approaches [22], and many centers consider it a gold standard method for both short and long-term eyelid closure [25].

Tarsorrhaphy can be partial or complete, and partial tarsorrhaphy can be central, medial, or lateral [21]. Various techniques have been introduced, which can be divided into permanent or temporary techniques [20,25]. Generally, temporary tarsorrhaphies last 4 to 6 weeks and are performed by suturing the two eyelids together using a non-absorbable suture (e.g., silk, nylon, prolene, and catgut) without creating any incisions or excisions [22,25,26]. Temporary tarsorrhaphies can be further subdivided into bolstered and non-bolstered variants, with bolstered tarsorrhaphy being the most common technique. Tarsorrhaphies that involve no incision or excision are sometimes referred to as blepharorrhaphies. Conversely, in permanent techniques, excision or incision of the eyelid margins is made then sutured together, resulting in the healing of the two parts together. The sutures are removed weeks later. Although permanent tarsorrhaphies are reversible and can be opened, they may cause eyelid distortion which is one of the main disadvantages [21,25].

Generally, tarsorrhaphy is an easy and quick procedure. However, challenges include discomfort and cosmetic dissatisfaction. Additionally, eye drop administration and examination are not possible in complete tarsorrhaphy [23]. While tarsorrhaphy is considered a safe procedure, it can be associated with several complications, including trichiasis, pain, failure due to suture erosion, keloid formation, pyogenic granuloma, and lash-line necrosis [23]. Of note, some studies consider short-term non-surgical techniques of eyelid closure as forms of tarsorrhaphy including using tape or adhesive glue to approximate the eyelids, or inducing levator paralysis with botulinum toxin injection [22,26].

Importantly, tarsorrhaphy remains a cornerstone procedure for ocular surface protection in cases of CEDs unresponsive to conservative therapy. Its temporary or permanent forms offer flexible and effective options, particularly in severe DES, exposure keratopathy, or NK.

#### 3.2.2. Botulinum Toxin Injection

Injection of botulinum neurotoxin (BoNT) to induce ptosis has a similar effect to tarsorrhaphy and is considered a type of non-surgical tarsorrhaphy [22,26]. Injection of BoNT into the upper margin of the superior tarsal plate or over the levator palpebrae superioris causes ptosis that typically lasts around 6–12 weeks. This approach is less invasive and can be employed as an initial step before considering more aggressive surgical interventions when treating DES, facial paralysis, NK, and persistent CED [21,24,27].

In addition to the aforementioned applications of BoNT, it is also used to treat oculoplastic disorders such as hemifacial spasm, upper eyelid retraction (UER), lagophthalmos, and entropion [27]. Interestingly, BoNT has a therapeutic role in both decreasing and increasing tear fluid [28]. In a prospective interventional study, Sahlin et al. demonstrated that paralysis of the orbicularis oculi muscle by BoNT injection into the medial lower eyelid decreases the lower lid’s horizontal movement, and injection into the medial upper lid weakens the upper lid ’s vertical movement and discrete retraction. A single dose of BoNT injected into both eyelids seems to reduce lacrimal drainage to 52% [29,30], and paralysis of the peri-canalicular orbicularis oculi muscles results in impaired apposition of the puncta which reduces lacrimal drainage and preserves tear fluid. Therefore, injection of BoNT into the medial eyelids can be beneficial in the treatment of DES [28,31]. Temporary tarsorrhaphy or botulinum toxin-induced ptosis has been shown to effectively protect the cornea during acute exacerbations of ocular surface inflammation in oGVHD [32].

Notably, BoNT injection provides a reversible, non-surgical alternative to tarsorrhaphy for managing OSDs. Its dual role in modulating eyelid position and tear drainage makes it particularly valuable in cases of DES, facial nerve dysfunction, and acute oGVHD-related inflammation.

#### 3.2.3. Upper Eyelid Loading

Upper eyelid loading is another type of eyelid narrowing procedure that enhances passive lid closure and increases the blink response by adding extra weight to the upper eyelid, thereby improving corneal coverage [33]. This method is mostly used in treating lagophthalmos, particularly paralytic lagophthalmos [34]. In these patients, paralysis of the orbicularis oculi muscle leads to lagophthalmos, decreased blinking, and impairment of the nasolacrimal pump. Disruption of the facial nerve may also impair tear secretion and contribute to ocular surface instability. These events disrupt the tear film and can cause CED and exposure keratopathy [35,36]. Eyelid loading has some advantages over tarsorrhaphy, including a better cosmetic appearance and less impact on the visual field [37,38].

Various materials have been suggested for eyelid loading, such as stainless steel, tantalum, gold, platinum, cartilage, and hyaluronic acid (HA) gel [39]. Gold implants, first introduced decades ago, remain widely used due to their favorable physical and biocompatible properties. Gold is dense and malleable, mostly unreactive to tissue, can be camouflaged under different skin colors, and does not interfere with magnetic resonance imaging (MRI) [39,40]. Although more expensive, platinum had a few advantages to gold: platinum is denser, so platinum implants could be smaller than gold implants, and platinum often has a better cosmetic outcome, a lower complication rate, and better biocompatibility [39,40]. One of the most common complications of gold implants is allergic reactions, which usually resolve after replacement with platinum [39]. However, while extremely rare, a few cases of tissue reaction toward platinum implants have been reported [41,42].

Eyelid loading is generally easy to perform and reversible without altering the eyelid anatomy [34,35]. Surgical techniques for eyelid loading differ by location of implant placement, which include intra-orbital, septal, pretarsal, and supra-tarsal placement [43]. Although pretarsal implants are most commonly used [43], supra-tarsal placement may be associated with lower complication rates [37,44]. Aside from placement location, additional surgical techniques include pretarsal fixation, retrograde pretarsal procedure, and combined pretarsal and direct levator fixation, which each offer different advantages and disadvantages. Therefore, it is advisable to decide on the proper method with careful consideration on a case-by-case basis [39].

One of the most important aspects of eyelid loading is predicting the implant’s ideal weight, as the success and complication rates largely depend on this factor [45]. Many methods for predicting the ideal weight have been suggested; however, the proper weight should be decided based on the patient’s anatomy, severity of lagophthalmos, and the location of the implant [39]. Complications associated with incorrect implant weight include ptosis, implant migration, bulging, eyelid distortion, extrusion, induced astigmatism, infection, inflammation, allergic reactions, and suboptimal eyelid closure [34,39,40,43].

Another material that can be utilized for eyelid loading is HA. Besides cosmetic usage, they are used for treating lagophthalmos, eyelid retraction and malposition, and orbital volume deficiency [46]. For treating lagophthalmos, HA can be delivered via either a transconjunctival or transcutaneous approach into the pretarsal or pre-levator aponeurotic region [46,47]. This method has a similar effect as weight implants but is quicker, less invasive, and easier to perform. Moreover, the effects of HA injection are temporary and reversible, typically lasting 6–12 months [47]. Therefore, it is a suitable option for patients for whom surgery is considered high-risk or when lagophthalmos is expected to be transient [46,48,49]. Although more challenging, HA injection can be used to correct residual lagophthalmos in patients who had undergone eyelid weight implantation with implants lighter than the ideal size [46].

HA injection is generally considered safe, with transient side effects such as edema, pain, ecchymosis, and erythema. However, vascular occlusion by HA can result in tissue necrosis and embolization, which are the most concerning complications [46,49,50].

Importantly, eyelid loading—whether via metallic implants or HA injection—offers an effective, reversible solution for paralytic lagophthalmos, improving eyelid closure and corneal protection. Tailoring the technique and material to the individual patient’s anatomy and clinical context is essential to minimize complications and optimize outcomes.

#### 3.2.4. Upper Eyelid Retractor Weakening

The levator muscle, responsible for the retraction of the upper eyelid, is divided into two parts: the anterior part forms the levator aponeurosis, while the posterior part is known as Müller’s muscle [51,52,53]. The balance between these two muscles and the protractor of the upper eyelid (the orbicularis oculi muscle) determines the position of the eyelid [51,54]. Therefore, changes in the strength or innervation of the retractors can lead to either ptosis or lid retraction. In conditions such as TED, which often present with UER, similar issues occur. Inflammation, fat infiltration, fibrosis in both Müller’s muscle and the levator aponeurosis, along with overactivity of the sympathetic innervation of Müller’s muscle, all seem to contribute to the development of UER [55,56,57]. As a result, some surgical techniques aim to correct UER by making anatomical changes to these two muscles.

Goldstein technique involves levator tendon reattachment to the orbicularis and sub-brow skin [58]. Henderson later proposed detaching Müller’s and levator attachments from the tarsal plate without reattachment, though outcomes were unpredictable [59,60]. Putterman and Urist introduced excision of Müller’s muscle with or without partial levator tenotomy, which was later refined by Chalfin to avoid nasal ptosis [61,62]. Koornneef’s full-thickness anterior blepharotomy offered favorable outcomes in moderate-to-severe UER by incising all layers above the tarsus and was subsequently modified by Hintschich et al. [63,64,65].

Various other techniques have been described that focus on weakening, lengthening, or disinserting Müller’s muscle and/or the levator aponeurosis using either anterior or posterior approaches [57,64,66]. However, none of these methods have proven to be superior [60,64]. The optimal surgical approach should be tailored to the patient’s condition and the surgeon’s expertise. Surgeons must be cautious of challenges such as avoiding over-correction and secondary ptosis, under-correction, contour defects, and eyelid crease asymmetry [55,57].

Although most of these procedures have been used primarily to treat thyroid-related UER, they have also been proven to be effective in managing UER caused by facial nerve palsy [54]. These techniques offer better cosmetic outcomes compared to tarsorrhaphy and do not reduce the visual field [54]. Furthermore, unlike eyelid implantation, these methods do not use alloplastic materials, are less costly, and avoid certain complications such as extrusion [67].

Notably, upper eyelid retractor weakening techniques offer a tailored, anatomically based approach to correcting eyelid retraction, especially in TED. These procedures provide functional and cosmetic advantages over alternatives like tarsorrhaphy or eyelid loading, though careful patient selection and surgical precision are key to avoiding complications.

#### 3.2.5. Lower Eyelid Retractor Weakening

The capsulopalpebral fascia and inferior tarsal muscle act as the primary retractors of the lower eyelid [51]. Similar to the upper eyelid, various procedures are available to manage lower eyelid retraction (LER), which can be classified into two main approaches: (1) Recession of the capsulopalpebral fascia, with or without the use of a spacer, and (2) A composite recession of both the orbital septum and the lateral horn of the inferior retractor. These surgeries can be performed using either an anterior or posterior approach, although the transconjunctival route is preferred [64,68]. Compared to UER surgery, the use of spacers is more common in LER surgeries, particularly in cases where the retraction exceeds 3 mm (severe retraction) [64]. Additionally, in instances of extreme LER, floor decompression can be beneficial [69].

Spacer grafts are used to lengthen the eyelid and to compensate for tissue loss. After the recession of the lower eyelid retractors, the graft is inserted beneath the tarsal plate. A suitable spacer graft should be biocompatible, readily available, resistant to contracture, and possess a degree of stiffness. Various types of autologous, allogenic, and alloplastic spacer grafts are available, including hard palate mucosa, donor sclera, tarsal conjunctiva, porous polyethylene, cartilage, dermis, and acellular tissue matrix [64,70]. While numerous studies have evaluated different types of spacer grafts, none have been proven to be superior and further research is needed [70,71].

**Figure 2:**
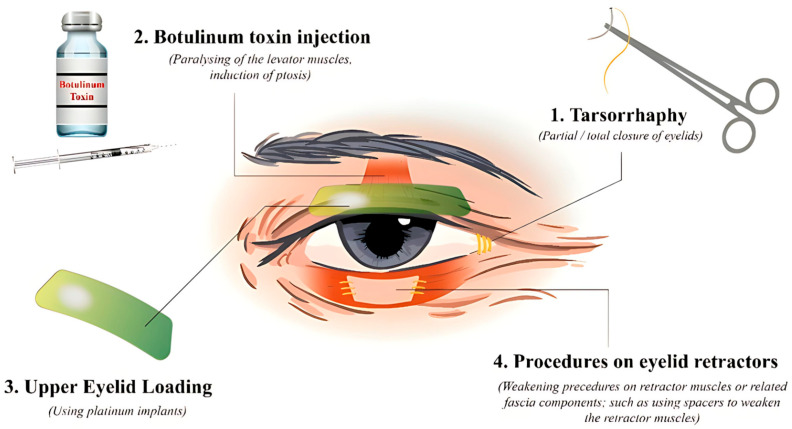
A brief illustrated description of eyelid fissure narrowing procedures.

Importantly, lower eyelid retractor weakening—often combined with spacer grafts—provides an effective surgical solution for moderate to severe LER. Selection of the spacer material and surgical technique should be individualized to each patient’s clinical context, as no single technique has shown clear superiority across all cases (Figure 2).

### 3.3. Corneal Neurotization

Corneal sensory innervation is provided by the first branch of the trigeminal nerve, known as the ophthalmic nerve. It activates the blinking reflex and tearing, and stimulates limbal stem cells thereby impacting corneal epithelial renewal [72,73]. Damage to corneal sensory innervation can lead to loss of tearing, decreased blinking reflexes, weakening of the corneal epithelium, and subsequent breakdown causing NK [74,75]. Numerous conditions can cause NK including herpetic infection, chemical or thermal burns, long-term use of contact lenses, trauma, neurosurgical or ocular operations, radiation exposure, and systemic diseases such as diabetes mellitus and multiple sclerosis [73,75,76,77,78,79,80,81,82]. Various surgical and non-surgical treatments are available for NK, including some of the aforementioned procedures such as AMT, conjunctival flap, tarsorrhaphy, and ptosis induced by BoNT injection [75,83,84], with surgical management mostly required in advanced stages of NK [21]. Most of these treatments aim to prevent disease progression, ameliorate NK symptoms, and subsequently restore corneal homeostasis by promoting corneal healing and regeneration [74,75,84]. However, a relatively new technique known as CN attempts to treat the underlying cause of NK [85]. Neurotization is a surgical method of transferring a healthy nerve segment to the injured tissue, aiming to restore sensory or motor innervation [86]. The first utilization of this technique for corneal innervation and treating NK was performed by Terzis et al. in 2009 in a case series study [72]. By repairing corneal sensation, CN seeks to restore corneal homeostasis, thereby promoting epithelial regeneration and healing [87]. CN has been successfully performed in both adults and children and is indicated for the treatment of NK, regardless of its underlying etiology [83].

Generally, there are three types of CN. The direct CN (DCN), introduced by Terzis et al., is performed through an incision and involves dissecting the supraorbital and supratrochlear nerves contralateral to the injured cornea. These nerves are subsequently tunneled across the nasal bridge to the upper lid crease and passed through the sub-Tenon’s space, where they are sutured to the ipsilateral perilimbal area of the affected cornea [72,83]. However, this technique has several disadvantages including a long operative time, extended recovery, visible scarring, alopecia at the incision site, and negative cosmetic outcomes [83]. Therefore, in 2017, Leyngold et al. in an experimental cadaveric feasibility study introduced a minimally invasive method known as minimally invasive CN (MICN), which involves harvesting the contralateral supraorbital nerve with the aid of endoscopy. This approach avoids the coronal incision, resulting in a smaller scar and shorter operative time [83,88]. In the indirect CN (ICN), first performed in 2014 by Elbaz et al., a conduit from the median cutaneous branch of the sural nerve is harvested and utilized for the procedure instead of the supratrochlear and supraorbital nerves. ICN can also be useful in treating bilateral NK, which is not possible with DCN [83,86].

**Figure 3:**
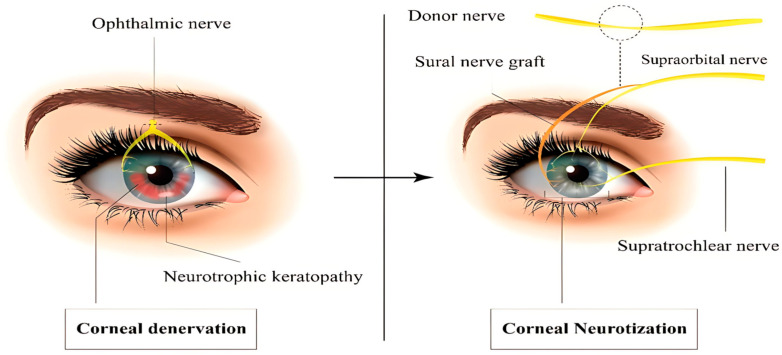
CN surgery, performed on the damaged denervated cornea.

Numerous studies have evaluated the therapeutic outcomes of CN and reported substantial curative effects. A systematic assessment of three studies including 58 patients demonstrated complete corneal healing in 99% of patients. However, CN was associated with a significantly longer healing time compared to other methods of NK treatment, such as AMT, nerve growth factor, and autologous serum, of an average of 117 days [74]. Another systematic review evaluating 17 studies and 60 patients found that corneal sensation improved for all patients undergoing CN, with 81% showing improvement in Mackie stage and 70% enhancement of visual acuity [89]. Although multiple studies reported similar therapeutic outcomes for DCN and ICN, the ICN technique demonstrated slightly superior improvements in corneal sensation [83,89]. However, CN is associated with some challenges. One is the identification of a proper nerve graft and fascicle separation, which requires substantial time and precision to avoid collateral damage [90]. Additionally, selecting a proper nerve graft is challenging due to considerations such as the sensory donor area, the distance between the donor area and the injured cornea, and the collectible size of the nerve graft [85]. Therefore, disturbance in the donor nerve dermatome sensation is a relative contraindication of CN. The main contraindication of CN is an active infection of the eye, especially herpetic keratitis. Active CED or corneal melting also pose moderate contraindications [87] (Figure 3).

Recent advancements in CN have introduced promising modifications to both DCN and ICN approaches, such as the use of acellular nerve allografts, which simplify the procedure while maintaining comparable outcomes to autografts [91,92]. Patient selection remains a key determinant of success, with early intervention yielding better corneal healing and sensory outcomes, particularly in pediatric patients who demonstrate faster neural regeneration [93,94]. Overall, CN demonstrates a high success rate, with postoperative corneal sensitivity improving from a mean of 2.7 mm to 36 mm on the Cochet–Bonnet aesthesiometer, and a reported recovery rate of up to 90% [85,91,95]. Nevertheless, the procedure poses technical challenges, including the need for specialized surgical expertise, variation in response based on graft technique (e.g., perilimbal vs. intrastromal), and a temporal mismatch between anatomical nerve regeneration and functional sensation recovery [85]. Moreover, long-term outcome data remain limited due to small sample sizes and variability in measurement techniques, highlighting the need for larger prospective trials and standardized reporting frameworks.

In summary, CN represents a promising intervention for NK by addressing the underlying nerve dysfunction and restoring corneal sensation. Despite its technical challenges and limited long-term data, CN has shown high success rates and continues to evolve with emerging techniques such as acellular grafts and minimally invasive approaches.

### 3.4. Amniotic Membrane Transplantation

AMT, a well-established therapeutic approach for OSDs, has gained increased attention in recent years due to advancements in its techniques. The amniotic membrane is rich in growth factors that facilitate wound healing and contains anti-inflammatory agents [96]. It can either be used as a graft or a patch. Patches are used when coverage is needed in reconstruction of the corneal epithelium. However, when an ocular morbidity is associated with deficient limbal stem cells, AMT has proven to be highly beneficial when it is used as a membrane graft prior to an upcoming limbal epithelial transplant [97,98]. Moreover, when used as a patch, the amniotic membrane enhances epithelial cell migration and differentiation, as well as basal cell adhesion [99].

Bulut et al. in a retrospective cohort study used a single-layer AMT with overlay technique in 13 eyes with infectious ulcerative keratitis, which yielded an almost 80% success rate within 30 days of follow-up [100]. Thatte reported favorable outcomes with single-layer AMT for CEDs and with multi-layer AMT for cases of corneal perforation, descemetocele, and stromal thinning. Moreover, optimal outcomes were observed when keratectomy was done with AMT in patients with Bullous keratopathy and chemical burns [101].

Gheorghe et al. in an interventional case series employed AMT in 28 patients presenting with ocular surface tumors, alkali burns, symblepharon, pterygium, or tumors of the cornea or conjunctiva. Their findings revealed clinically meaningful improvements in the majority of patients. However, AMT was less effective in cases of tumors or older burns [102]. Similarly, Muraine et al. in a prospective interventional case series reported favorable outcomes in 80% of 31 eyes treated with AMT. They further evaluated AMT in eight patients with persistent bullous corneal dystrophies, noting superior outcomes when the transplant was sutured anterior to the limbus compared to placement beyond the limbus onto the conjunctiva. Furthermore, isolated AMT without concomitant limbal stem cell transplantation was found to provide insufficient healing in cases of chemical burns. Therefore, in such cases, stem cell transplantation is suggested after AMT. Complications associated with AMT, such as neovascularization and graft detachment, were documented in 12 of the 31 eyes. Overall, AMT yielded more favorable outcomes in the treatment of chronic ulcers with or without limbal involvement compared to chemical burns, persistent bullous corneal dystrophies, and symblepharon [103]. Tabatabaei et al. in a randomized clinical trial study reported favorable outcomes using double-layered AMT combined with antibiotic treatments compared to isolated antibiotic therapy for patients with bacterial keratitis. They reported reduced neovascularization, scarring, and inflammation after one month of treatment [104].

Prabhasawat et al. in a prospective interventional case series found that when AMT is executed using a multilayer technique, optimal corneal thickness can be achieved even in cases of corneal perforation. Nonetheless, consistent with the aforementioned studies, the success rate for all patients irrespective of the degree of corneal stromal layer damage was reported to be up to 80%. Complications post-AMT were observed in five patients including infection, NK, and one case of perforation with no instances of graft rejection reported [105]. Tseng et al. in a prospective interventional case series used intraoperative mitomycin C shortly before the AMT in the deep fornix in patients with severe burns, which resulted in better healing of the ocular surface, reduction of the inflammation, and tear meniscus enhancement [106]. AMT has been effectively utilized in progressive corneal ulcers associated with oGVHD, contributing to epithelial healing and inflammation control [107,108].

In conclusion, AMT is a versatile and widely adopted technique in the management of various OSDs, with applications ranging from CEDs to corneal perforations and chemical burns. Its success largely depends on the indication, technique of application, and the presence of LSCD, and it has shown particularly favorable outcomes in chronic ulcers and oGVHD-associated epithelial disease.

### 3.5. Conjunctival Flap Surgery

Conjunctival flap surgery remains a valuable therapeutic option for patients with chronic noninfectious OSDs that are refractory to medical therapy. In conditions such as NK, bullous keratopathy, and severe DES, conjunctival flaps play a crucial role in restoring epithelial integrity, relieving pain, and preserving the globe in eyes with limited visual potential. By supplying vascularized tissue and creating a mechanical barrier, conjunctival flaps not only halt stromal degradation but also reduce the burden of frequent medication use and enhance cosmetic appearance [109,110]. For instance, in bullous keratopathy, conjunctival flap surgery eliminates painful bullae and provides a stable ocular surface, substantially improving patients’ quality of life [109,110]. In neuroparalytic cases, the vascular protection offered by the flap helps resist ulceration and perforation due to the presence of anticollagenolytic substances from the bloodstream [109,111].

While alternative surgical techniques such as AMT and keratoplasty have become more common, conjunctival flap surgery retains specific advantages in select cases. It is especially useful in eyes with limited access to donor tissue or patients with systemic contraindications to more invasive surgeries. Furthermore, its role in relieving pain, stabilizing the ocular surface for prosthetic devices, and preserving the eye cosmetically in blind or non-seeing eyes is well established [109,110]. The development of refined techniques such as pedicled or advancement flaps has improved surgical outcomes and minimized complications. In carefully selected patients, these modern adaptations make conjunctival flap surgery a safe, effective, and resource-efficient strategy for long-term ocular surface stabilization [109,112,113]. Gundersen flaps have been applied in oGVHD patients with impending perforation, often in combination with tectonic keratoplasty, and have shown promising outcomes [114,115].

In summary, conjunctival flap surgery remains an important tool in the surgical management of refractory noninfectious OSDs. Its ability to restore surface integrity, relieve pain, and preserve the globe makes it particularly valuable in selected patients, especially when other advanced interventions are contraindicated or unavailable.

### 3.6. Salivary Gland Transplantation

Minor salivary glands are responsible for nearly half of salivary secretion and can be transplanted to the posterior lamella of the eye to enhance lubrication. Minor salivary gland transplantation (MSGT) has proven effective in treating severe DES by continuously lubricating the ocular surface with saliva instead of tears. Results last up to 10 years post-transplantation, and this technique requires no vascular anastomosis and utilizes readily available grafts, making it a cost-effective procedure [116]. In cases of mild to moderate DES, pharmaceutical agents like eye drops are the primary treatment. For persistent severe disease, submandibular gland transplantation (SMGT) is recommended. SMGT has been shown to improve tear break-up time (TBUT), corneal fluorescein staining, and rose Bengal staining tests [117,118]. Autologous sublingual gland tissue has demonstrated minimal efficacy in DES treatment, with limited reported cases and concerns about necrosis limiting its use [119].

Vazirani et al. in a retrospective case series employed a modified MSGT technique, fixing a complex of mucosa, gland, and muscle tissue to the upper bulbar surface and the superior rectus muscle in patients with SJS and mucous membrane pemphigoid (MMP). Post-MSGT, best-corrected visual acuity (BCVA) improved from 20/500 to 20/125, accompanied by improvements in DES symptoms. This improvement likely resulted from overall enhancements in ocular surface health. Enhanced Schirmer scores and conjunctival and corneal staining scores were also observed, with no additional serious complications [120]. However, a retrospective cohort study by Su et al. found suboptimal results with MSGT compared to SMGT for severe DES cases. Post-transplantation, no improvements in BCVA and TBUT were observed, with only 60% symptom relief. However, when MSGT was used in patients with less severe DES, outcomes were more favorable, with improvements in TBUT and up to 80% symptom relief. Thus, MSGT appears most beneficial when chosen as a treatment option for patients with non-end-stage DES and without cicatrizing etiologies [116]. Another prospective interventional study by Su et al. evaluated the quality of life in patients with DES after SMGT, with anastomosis of the facial artery and vein to the superficial temporal artery and Wharton’s duct to the upper lateral conjunctival fornix. The study demonstrated notable enhancements in patients’ symptoms, routine daily tasks, satisfaction, and emotional well-being. These results, along with the aforementioned studies, suggest that symptom relief post-transplantation can reach up to 80% but can vary significantly. It is important to counsel patients preoperatively about the possibility of incomplete symptom resolution following transplantation [118].

SGT is not applicable in cases of Sjögren’s syndrome and xerostomia due to insufficient saliva production (flow rate < 0.3 g/m) and potential worsening of symptoms in the donor. SGT is also not recommended in cases of multiple gland dysfunctions [121,122]. Indications for transplant are as follows: (1) refractory symptoms with no improvements despite treatment with medications, (2) a Schirmer’s test value of less than 2 mm, (3) TBUT < 5 s, and (4) positive fluorescence staining [121,122].

Complications of SMGT include epiphora, excessive saliva production during the first three months post-transplantation, potentially causing duct obstruction and treatment failure, and duct inflammation due to excessive production [123].

In conclusion, SGT—particularly MSGT and SMGT—offers a promising surgical approach for managing severe, treatment-resistant DES. While MSGT is better suited for non-end-stage DES without cicatrization, SMGT demonstrates superior results in advanced cases but requires careful patient selection and counseling due to its complexity and potential complications.

Table 1 summarizes the key oculoplastic interventions and their clinical applications (Table 1).

**Figure 4:**
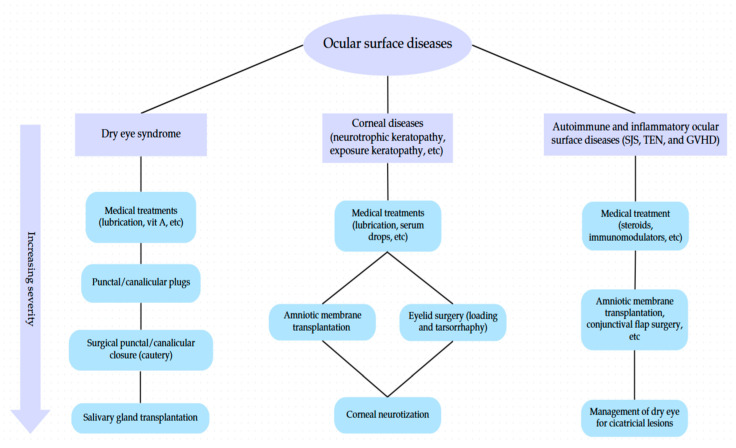
Flowchart illustrating treatment pathways for OSDs based on etiology and severity.

To enhance clinical applicability, a flowchart was developed to outline surgical decision-making pathways for OSDs, stratified by disease type and severity (Figure 4).

### 3.7. Future Direction

Recent advances in regenerative medicine have opened new avenues for the management of OSDs, particularly in conditions where traditional therapies have limited efficacy. In LSCD, regenerative strategies aim to restore a functional corneal epithelium through transplantation of autologous or allogenic cells. Techniques such as conjunctival limbal autografting (CLAU), cultivated limbal epithelial transplantation (CLET), and simple limbal epithelial transplantation (SLET) have demonstrated varying degrees of clinical success, with CLET offering reduced donor site morbidity due to minimal biopsy requirements [124,125,126,127]. More recent innovations include the use of bioengineered scaffolds such as fibrin-agarose composites and cell-laden contact lenses, which mimic native corneal architecture and facilitate in vivo cell expansion [128,129]. Additionally, other sources of epithelial cells—such as oral mucosa, conjunctival epithelium, and stem cells derived from hair follicles, dental pulp, or induced pluripotent stem cells (iPSCs)—are promising but still experimental options for treating severe bilateral disease [130,131,132,133].

In the context of DES, particularly in immune-mediated conditions like chronic oGVHD, regenerative treatments aim to restore tear film homeostasis and suppress inflammation. Adipose-derived mesenchymal stem cells (ADMSCs) have shown potential in both local (lacrimal gland) and systemic (intravenous) administration, improving Schirmer test scores and reducing symptom burden in patients with severe aqueous deficiency [134,135,136,137,138]. Mesenchymal stem cell-derived exosomes (MSC-exo) have also demonstrated therapeutic promise when used topically, owing to their immunomodulatory and anti-inflammatory properties [139]. While these approaches remain investigational, early results are encouraging and point toward a future in which biologic therapies complement or even replace traditional ocular surface interventions.

In summary, regenerative medicine holds significant promise for transforming the treatment landscape of OSDs. Techniques such as limbal stem cell transplantation, bioengineered scaffolds, and stem cell-derived therapies offer innovative, targeted solutions that may overcome limitations of conventional surgical methods, especially in complex or refractory cases.

## 4. Conclusions

Reconstructive ocular surface interventions are crucial in managing OSDs when non-invasive therapies prove insufficient. Procedures like AMT, eyelid fissure narrowing techniques, and conjunctival flap offer varying degrees of success in alleviating symptoms and restoring ocular function. In particular, conditions such as oGVHD may require advanced surgical approaches, including punctal cauterization and multilayered AMT. While some surgeries provide substantial relief, others may yield more modest improvements. However, it is essential to carefully consider the indications, contraindications, and potential complications of each procedure to optimize patient outcomes. By tailoring surgical approaches to individual patient needs, clinicians can effectively employ these interventions to enhance quality of life and ocular health for individuals with OSDs.

## Figures and Tables

**Table 1:** Comparative summary of oculoplastic surgical interventions for OSDs.

Intervention	Main Indications	Advantages	Limitations/Complications	Comparison Notes
Punctal Occlusion (Punctal plug, Canalicular plug, and Surgical occlusion)	DES, Sjögren’s, SJS, NK, SLK, contact lens-related dryness	Non-invasive, improves tear retention, can serve as drug delivery system	Plug loss/extrusion, irritation, canaliculitis, biofilm, epiphora	Compared to surgery, plugs are easier to apply but have higher extrusion rates.
Tarsorrhaphy	Persistent CED, exposure keratopathy, facial nerve palsy	Simple, protective, effective in both short- and long-term settings	Cosmetic dissatisfaction, prevents eye drop use in complete closure	More invasive than BoNT or eyelid loading, but more durable in severe exposure.
Botulinum Toxin Injection	DES, epiphora due to NLDO, hemifacial spasm, eyelid retraction	Minimally invasive, dual role in tear modulation	Short duration (6–12 weeks), potential for undesired ptosis or undercorrection	Less invasive than tarsorrhaphy; suitable for temporary disease.
Upper Eyelid Loading	Paralytic lagophthalmos, facial nerve palsy, eyelid retraction	Cosmetically favorable, reversible, avoids visual field restriction	Implant migration, extrusion, astigmatism, allergic reaction (esp. gold)	Better cosmesis and vision preservation than tarsorrhaphy; more durable than BoNT.
Upper Eyelid Retractor Weakening	Upper eyelid retraction (TED, facial palsy)	Preserves cosmesis and visual field, avoids foreign implants	Contour defects, asymmetry, ptosis/undercorrection, requires surgical expertise	More cosmetic and cost-effective than tarsorrhaphy or implants
Lower Eyelid Retractor Weakening	LER > 3 mm, severe lagophthalmos	Effective for severe cases with use of spacers, transconjunctival route preferred	Spacer-dependent results, graft-related issues (contracture, extrusion)	Better than tarsorrhaphy for functional/aesthetic outcomes in lower lid
Corneal Neurotization	NK unresponsive to conservative therapy	Addresses root cause by restoring corneal sensation, improves long-term epithelial healing	Technically demanding, longer healing time, risk of donor nerve disturbance	Superior in etiology-targeted treatment compared to AMT/tarsorrhaphy; MICN and ICN minimize invasiveness
Amniotic Membrane Transplantation	Persistent epithelial defects, chemical burns, LSCD, corneal ulcers	Anti-inflammatory, promotes epithelialization, useful in multilayer for stromal thinning	Risk of neovascularization, detachment, less effective in older burns or tumors	Preferred over conjunctival flap for healing but may require adjunctive stem cell transplant in LSCD
Conjunctival Flap Surgery	Deep ulcers, neurotrophic/infectious keratitis, non-healing corneal perforations	Effective tectonic support in absence of grafts, avoids evisceration, accessible technique	Reduced visual potential, flap retraction, cysts, not suitable in Mooren’s or autoimmune ulcers	Useful when AMT unavailable; inferior in optical outcomes but superior in structural preservation
Salivary Gland Transplantation	Severe refractory DES (non-Sjögren), especially with cicatricial disease (e.g., SJS, MMP)	Long-term lubrication, cost-effective, high symptom relief rates (~80%)	Contraindicated in xerostomia/Sjögren; excess secretion, duct issues may occur	Provides continuous lubrication unlike drops/plugs; best for end-stage DES where other methods fail

## Data Availability

The data supporting the findings of this study are available from the corresponding author, M.S., upon reasonable request.
